# Artificial *Erythrina* Alkaloids from Three *Erythrina* Plants, *E. variegata*, *E. crista*-*galli* and *E. arborescens*

**DOI:** 10.1007/s13659-020-00235-3

**Published:** 2020-03-04

**Authors:** Bing-Jie Zhang, Jing Wu, Mei-Fen Bao, Fang Wang, Xiang-Hai Cai

**Affiliations:** 1 State Key Laboratory of Phytochemistry and Plant Resources in West China, Kunming Institute of Botany, Chinese Academy of Sciences, Kunming, 650201 China; 2 University of Chinese Academy of Sciences, Beijing, 100049 China; 3 Yunnan Key Laboratory of Natural Medicinal Chemistry, Kunming, 650201 China

**Keywords:** Fabaceae, *Erythrina variegata*, *E. crista*-*galli*, *E. arborescens*, Artificial products, *Erythrina* alkaloids

## Abstract

Fourteen unprecedented artificial *Erythrina* alkaloids were isolated from the *Erythrina variegata*, *E. crista*-*galli* and *E. arborescens* (Fabaceae). The structures of these alkaloids were determined by spectroscopic analyses. Their possible formations were proposed. All isolated compounds showed no cytotoxicity and hypoglycemic activity at cell screening bioassay.

## Introduction

**Fig. 1:**
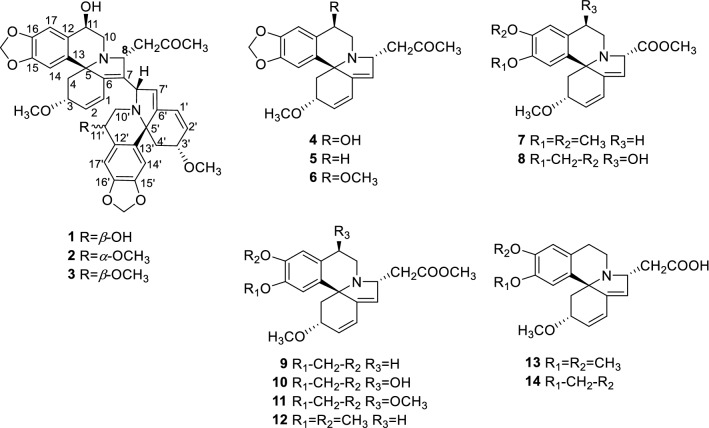
Chemical structures of 14 artificial *Erythrina* alkaloids

The *Erythrina*-type alkaloids with 6/5/6/6 spirocycle systems and stable 5*S*-chiral center are derived from two tyrosine units via oxidative coupling and intramolecular rearrangement. Since the first phytochemical research on *Erythrina* alkaloid in 1930s [1], the total number now stands at well over 110 alkaloids reported from plants of *Erythrina* genus [2]. Bioassay screening of these alkaloids showed anxiolytic-like actitivity [3], induced sleep [4], anticonvulsant actitivity [5], neuronal nicotinic acetylcholine receptor antagonism [6], leishmanicidal [7], anticataract [7], and antifeedant activity [8]. Our previous research disclosed natural dimeric [9, 10] and trimeric [11] *Erythrina* alkaloids, alkaloidal glucosides [10], and complex monomers [12–14]. However, during the extraction and separation of *Erythrina* alkaloids, some artificial products would be produced. Consideration of the NMR spectra characteristics and potential activities of *Erythrina* alkaloid, we here systematically summarized these artifacts from *E*. *arborescens*, *E*. *crista*-*galli* and *E*. *variegata* (Fig. 1). Their cytotoxicity against three cancer cells and hypoglycemic activity on 3T3-L1 myoblasts cell were screened. This paper will describe their isolation, structure determination and possible mechanism of formation.

## Results and Discussion

Alkaloid **1** was obtained as white amorphous powder. Its IR absorption bands at 3441, 1640, 1503, 1480 cm^−1^ indicated the presence of the hydroxyls and aromatic rings. Moreover, the UV absorptions at 204, 239 and 290 nm indicated a tetrahydroisoquinoline chromophore [15]. These spectra were consistent with the characteristics of an *Erythrina* alkaloid. Alkaloid **1** had a molecular formula C_39_H_40_N_2_O_9_ as established by the HRESIMS *m/z* at 681.2811 [M+H]^+^, together with the ^1^H and ^13^C NMR spectroscopic data. In the ^1^H NMR spectrum of **1** (Table 1), four aromatic singlet proton (*δ*_H_ 7.08, 7.01, 6.74 × 2), two pairs of olefin [*δ*_H_ 7.16 (*dd*, *J* = 10.2, 4.2 Hz), 5.92 (*d*, *J* = 10.2 Hz), and 6.56 (*dd*, *J* = 10.2, 2.4 Hz), 6.06 (*d*, *J* = 10.2 Hz)], two methylenedioxy group (*δ*_H_ 5.96, 5.94, 5.93 and 5.92), and two methoxyl group (*δ*_H_ 3.29 and 3.24) signals indicated that **1** might be an *Erythrina* alkaloid dimer. In comparison with the reported dimer, erythrivarine A [9], alkaloid **1** had three more signals at *δ*_C_ 208.1, 47.5 and 31.2 in the ^13^C NMR spectra and 56 mass units higher in molecular weight, which showed an extra 2-oxopropyl group in **1**. In the HMBC spectrum, correlations of *δ*_H_ 4.20 (H-8) with *δ*_C_ 53.9 (C-10), *δ*_C_ 136.2 (C-7) and *δ*_C_ 208.1 (C=O) indicated the 2-oxopropyl group at C-8. Further analysis of the 2D NMR revealed that the other parts of compound **1** were consistent with those of erythrivarine A.

**Table 1:** ^1^H and ^13^C NMR spectroscopic data for **1**–**3** in acetone-*d*_6_ (*δ* in ppm and *J* in Hz)

Enrty	**1**		**2**		**3**	
	*δ*_H_	*δ*_C_	*δ*_H_	*δ*_C_	*δ*_H_	*δ*_C_
1	7.16, dd (10.2, 4.2)	124.6 d	7.16, d (10.2)	124.8 d	7.06, overlap	124.7 d
2	5.92, d (10.2)	132.1 d	6.00, d (10.2)	132.2 d	6.00, d (10.2)	132.2 d
3	3.68, m	76.9 d	3.73, m	76.8 d	3.73, m	77.0 d
4	2.38, dd (11.4, 6.0)	43.6 t	2.44, dd (10.8, 6.0)	42.9 t	2.40, dd (10.8, 6.0)	43.0 t
	1.68, t (11.4)		1.69, t (10.8)		1.70, t (10.8)	
5		69.2 s		68.9 s		69.2 s
6		139.6 s		139.7 s		138.2 s
7		136.2 d		136.8 d		135.6 d
8	4.20, m	69.7 d	4.30, m	68.3 d	4.23, br s	69.2 d
10	3.48, dd (11.4, 5.4)	53.9 t	3.34, dd (11.4, 4.8)	50.7 t	3.47, dd (11.4, 4.8)	53.2 t
	2.51, overlap		2.77, br d (11.4)		2.61, overlap	
11	4.65, m	64.7 d	4.66, m	64.7 d	4.75, m	64.8 d
12		134.1 s		133.9 s		133.9 s
13		132.2 s		132.6 s		132.6 s
14	6.74, s	106.1 d	6.73, s	105.8 d	6.90, s	106.1 d
15		147.7 s		147.3 s		147.3 s
16		147.2 s		147.3 s		147.3 s
17	7.01, s	108.7 d	7.01, s	108.6 d	7.06, s	107.3 d
OCH_2_O	5.96, s	101.8 t	5.96, s	101.8 t	5.96, s	102.0 t
	5.94, s		5.95, s		5.95, s	
3-OCH_3_	3.29, s	56.3 q	3.26, s	56.2 q	3.25, s	56.2 q
11-OH					4.63, d (5.4)	
CH_2_COCH_3_	3.21, overlap	47.5 t	3.42, m	47.1 t	3.35, m	47.5 t
	2.71, m		2.71, m		2.64, m	
CH_2_COCH_3_		208.1 s		207.8 s		208.2 s
CH_2_COCH_3_	2.14, s	31.2 q	2.13, s	31.0 q	2.13, s	31.2 q
1′	6.56, dd (10.2, 2.4)	125.5 d	6.55, dd (10.2, 1.8)	125.5 d	6.54, dd (10.2, 1.8)	125.7 d
2′	6.06, d (10.2)	133.6 d	6.06, d (10.2)	133.6 d	6.09, d (10.2)	133.6 d
3′	3.32, m	76.8 d	3.93, m	76.8 d	4.06, m	76.6 d
4′	2.46, dd (11.4, 5.4)	41.6 t	2.44, dd (10.8, 6.0)	41.6 t	2.49, dd (10.8, 6.0)	40.9 t
	1.79, t (11.4)		1.79, t (10.8)		1.77, t (10.8)	
5′		68.2 s		68.4 s		68.3 s
6′		142.8 s		143.2 s		143.4 s
7′	5.58, s	127.3 d	5.57, s	127.4 d	5.58, s	127.4 d
8′	4.92, s	66.4 d	4.90, s	64.6 d	4.92, s	64.5 d
10′	3.36, dd (13.2, 4.8)	50.5 t	3.42, dd (13.2, 4.0)	47.0 t	3.21, m	43.2 t
	2.83, overlap		2.83, dd (13.2, 5.0)		3.15, m	
11′	4.78, m	64.9 d	4.24, s	74.7 d	4.00, s	74.5 d
12′		132.5 s		129.2 s		129.2 s
13′		133.4 s		132.6 s		132.6 s
14′	6.74, s	105.9 d	6.78, s	106.0 t	6.75, s	105.8 d
15′		147.3 s		147.3 s		148.4 s
16′		147.2 s		147.3 s		147.3 s
17′	7.08, s	106.8 d	6.92, s	108.1 d	7.37, s	110.3 d
OC’H_2_O	5.93, s	101.7 t	5.95, s	101.8 t	5.95, s	101.8 t
	5.92, s		5.94, s		5.94, s	
3′-OCH_3_	3.24, s	56.2 q	3.29, s	56.2 q	3.35, s	56.3 q
11′-OCH_3_			3.54, s	58.1 q	3.36, s	58.0 q

^*1*^*H NMR* recorded at 600 MHz, ^*13*^*C NMR* recorded at 150 MHz

Alkaloids **2** and **3** showed the same molecular formula C_40_H_42_N_2_O_9_ as established by HRESIMS (*m/z* 695.2968 [M+H]^+^ in **2**; *m/z* 695.2970 [M+H]^+^ in **3**). In the ^1^H and ^13^C NMR spectra, the chemical shifts of **2** and **3** showed good agreement with those of **1**, except those signals around the C-11′ position (C-10′/11′/12′). The C-11′ carbon of **1** was resonated at *δ* 64.9, however, signals of the same carbon were observed at *δ* 74.7 and *δ* 74.5 in **2** and **3**, respectively. In addition, an extra methoxyl group (*δ*_H_ 3.54 in **2**, *δ*_H_ 3.36 in **3**) was observed, which indicated that both **2** and **3** had a methoxyl group at the C-11′ position instead of a hydroxyl group in **1**. Chemical shifts of H-10′, H-11′ and H-17′ protons (Table 1) of **2** and **3** implied the configuration of 11′-OCH_3_ in **2** and **3** were different. In the ROESY spectrum, the NOE correlation of H-3′*β*/H-4′*β* and H-11′/H-4′*α* in **3** suggested its 11′-OCH_3_ was in *β*-orientation. The NOE correlation of H-11′/H-4′*β* in **2** suggested its 11′-OCH_3_ was in *α*-orientation.

The HRESIMS of **4** showed the pseudomolecular ion at *m/z* 370.1651 [M+H]^+^ (calc. for C_21_H_23_NO_5_, 370.1652). The ^13^C NMR spectrum of **4** showed a methine at 64.7 ppm, which indicated the existence of hydroxy substituent. The HMBC correlation between *δ*_H_ 7.05 (H-17) with *δ*_C_ 64.7 suggested the hydroxy at C-11. Through detailed comparison of the 1D and 2D NMR spectrum, **4** was basically the same as erythranine [16] except for the 2-oxopropyl substituent (*δ*_C_ 207.9, 47.8 and 30.8). The HMBC spectrum showed correlations from *δ*_H_ 3.98 (H-8) to *δ*_C_ 52.6 (C-10), *δ*_C_ 127.6 (C-7) and *δ*_C_ 207.9 (C=O) disclosed **4** to be 8-(2-oxopropyl)-erythranine.

Alkaloid **5** displayed a hydrogen adduct ion at *m/z* 354.1709 [M+H]^+^ (calc. for C_21_H_23_NO_4_, 354.1707). The 1D NMR spectroscopic data of compound **5** were similar to those of **4** except for the following differentiations: in the ^1^H NMR spectrum, the signal displayed at *δ*_H_ 4.72 in **4** which was assigned to the active hydrogen in the hydroxy was disappeared in compound **5**. Correspondingly, the methine signal at *δ*_C_ 64.7 (C-11) in compound **4** was replaced with a methylene (*δ*_C_ 26.0) in **5**. Thus, compound **5** was an analogue of **4** without the hydroxy moiety and determined to be 8-(2-oxopropyl)-erythraline.

The HRESIMS of **6** gave a hydrogen adduct ion at *m/z* 384.1806 [M+H]^+^, indicative of a molecular formula of C_22_H_25_NO_5_. In comparing with those of **4**, the ^1^H NMR spectrum of **6** gave signal of an addional methyoxyl group (*δ*_H_ 3.55, s, 3H), and its ^13^C NMR spectrum showed an downfield chemical shift *δ*_C_ 74.8. These findings suggested the C-11 of **6** was substituted by a methoxy rather than a hydroxy. Thus, the structure of **6** was determined to be 8-(2-oxopropyl))-11-methoxy-erythraline.

The molecular formula of **7** was determined to be C_21_H_25_NO_5_ from the HRESIMS *m/z* at 394.1626 [M+Na]^+^. Its ^1^H NMR spectrum showed two aromatic singlet protons (*δ*_H_ 6.84 and 6.76), three conjugate olefin signals (*δ*_H_ 6.60, 6.10 and 5.67), and four methoxy groups (*δ*_H_ 3.28, 3.70, 3.78 and 3.97). The ^13^C NMR spectrum of **7** showed three methylenes (*δ*_C_ 24.6, 43.3, 44.4), two methines (*δ*_C_ 76.9 and 70.5) and a carbonyl (*δ*_C_ 172.1). These data suggested **7** might be a carbomethoxyl derivative of erysotrine [17]. The HMBC correlations from *δ*_H_ 3.78 (OCH_3_) and *δ*_H_ 4.33(H-8) to *δ*_C_ 172.1 (C=O) assigned the carbomethoxy at C-8. The molecular formula of **8** was determined to be C_20_H_21_NO_6_ from the HRESIMS *m/z* at 372.1444 [M+H]^+^. The ^1^H and ^13^C NMR the structural pattern of **8** was identical to that of **5**, and the additional carbomethoxyl moiety was identical to that of **7**. Accordingly, the structures of **7** and **8** were determined to be 8-carbomethoxyerysotrine and 8-carbomethoxyerythranine, respectively.

Alkaloid **9** showed molecular ion peaks at *m/z* 370.1652 [M+H]^+^, suggesting the molecular formulae C_21_H_24_NO_5_. In comparing with compound **5**, the ^1^H and ^13^C NMR signal of *δ*_H_ 2.13 (CH_3_) and *δ*_C_ 207.7(CH_2_COCH_3_) in **5** were changed to *δ*_H_ 3.61 (OCH_3_) and *δ*_C_ 172.5(CH_2_COOCH_3_) in **9**, respectively. The remaining NMR data were almost identical to those of **5**. Thus, the structure of **9** was determined to be 8-acetatemethoxyerythraline.

The molecular formulas of compounds **10** and **11** were deduced to be C_21_H_23_NO_6_ and C_22_H_25_NO_6_ from the HRESIMS at *m/z* 386.1599 [M+H]^+^ and 400.1758 [M+H]^+^, respectively. The ^1^H and ^13^C NMR data of both **10** and **11** are very similar to those of **9** except that the methylene signal was replaced by an oxymethine signal at the C-11 position. Further, in the ^13^C NMR, the signal for C-11 appeared at 64.8 and 74.7 ppm for compounds **10** and **11**, similar to that of **4** and **6**, respectively. Thus, **10** was identified as 8-acetatemethoxyerythranine. **11** had an extra methoxy and was identified as 8-acetatemethoxy-10*β*-methoxyerythraline.

The molecular formula **12** was established as C_22_H_27_NO_5_ based on the HRESIMS *m/z* = 386.1964 [M+H]^+^. From the ^1^H and ^13^C NMR data, the structure of **12** was very similar to **9** except for the replacement of methylenedioxy group by two methoxys at C-15 and C-16. This was confirmed from the HMBC and HSQC spectra. Alkaloid **12** was thus identified as 8-acetatemethoxyerysotrine.

The HRESIMS *m/z* at 394.1628 [M+Na]^+^ of **13** assigned the molecular formula to be C_21_H_25_NO_5_, 58 mass units higher than that of erysotrine. Its ^13^C NMR spectrum gave an additional methylene (*δ*_C_ 34.9) and a carbonyl (*δ*_C_ 172.3) signals, indicating the existence of an acetyl group. The HMBC correlations from *δ*_H_ 2.51 (CH_2_CO) and *δ*_H_ 4.14 (H-8) to *δ*_C_ 172.3 (C=O) suggested that the acetyl group was located at C-8. Accordingly, the structure of **13** was determined to be 8-acetylerythsotrine. The molecular formula of **14** was dirermined to be C_20_H_21_NO_5_ by the HRESIMS *m/z* at 378.1313 [M+Na]^+^. The 1D NMR spectrum gave signals similar to that of erythraline expect for the replacement of a methylene by an acetyl group (*δ*_C_ 35.4 and *δ*_C_ 172.3). In the HMBC spectrum, the correlations from *δ*_H_ 4.07 (H-8) to *δ*_C_ 172.3 (COOH) and *δ*_H_ 2.74 (CH_2_COOH) to *δ*_C_ 65.4 (C-8) and *δ*_C_ 172.3 (COOH) confirmed that **14** was an 8-acetyl derivative of erythraline.

**Fig. 2:**
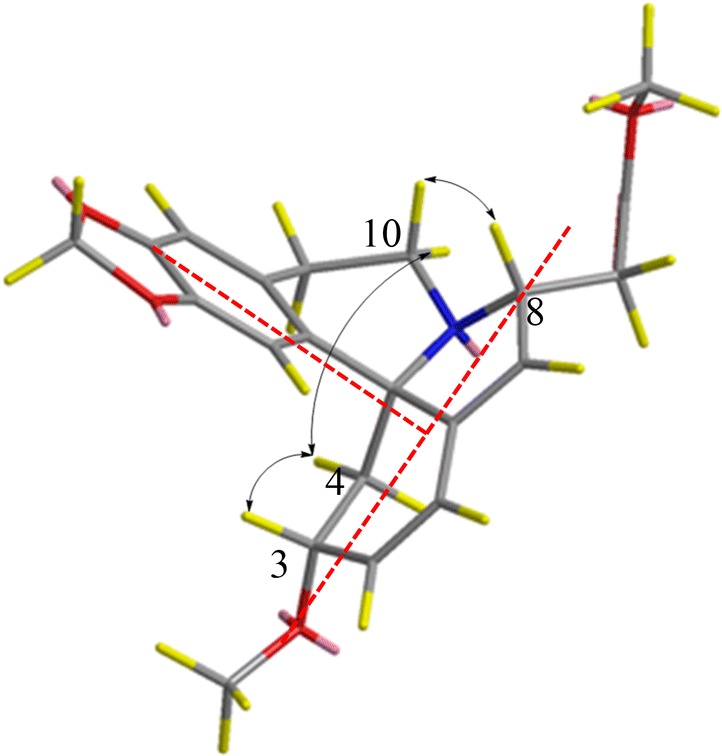
Selected NOE interaction of alkaloid **9**

The configurations of H-8 for compound **1**–**14** were determined to be *β* based on ROESY experiments with correlations of H-3*β*/H-4_eq_, H-4_eq_/H-10_ax_ and H-10_eq_/H-8 (Fig. 2). Further, together with 5*S*-configuration in all *Erythrina* alkaloids [18], so absolute configuration of alkaloids **1**–**14** could be determined.

**Fig. 3:**
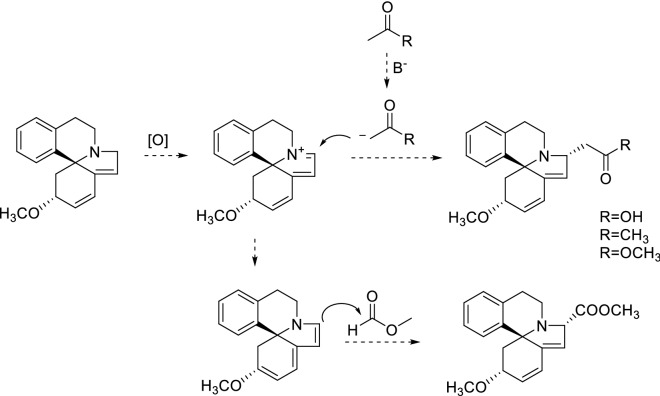
Possible formation of two typically artificial *Erythrina* alkaloids

Since N-containing compounds were main candicates of anticancer and hypoglycemic drugs, so alkaloids **1**–**14** were evaluated for their cytotoxicity against human A-549 lung cancer, SGC-7901 gastric cancer, and HeLa cell lines using the MTT method. In addition, their hypoglycemic activity on 3T3-L1 myoblasts cell were screened. Unfortunately, none of them showed positive activity. Alkaloids **1**–**14** possessed acetonyl, acetyl methyl, acetate, or methyl formate groups, which indicated they were artificial products. Without considering the artifitial units, these alkaloids are known. Duing the extraction and isolation, methanol, acetone, petroleum ether, especial ethyl acetate, were used as solvents. Accordingly, acetone and residual of acetic acid, methyl acetate and methyl formate in above solvents would become reaction reagents. Alkaloids **1**–**6** and **9**–**14** were formed firstly through an iminium immediate by oxidation, then by nucleophilic attack from carbanion of acetone, acetic acid, and methyl acetate in base condition. On the other hand, the iminium immediate could be tautomerized to inmine and attacked to methyl formate, generating the carbomethoxy substitued products (**7**–**8**) (Fig. 3).

## Experimental Section

### General Experimental Procedures

Optical rotations were measured with a Jasco p-1020 digital polarimeter. UV spectra were recorded on a Shimadzu 2401PC spectrophotometer. IR spectra were obtained on a Bruker Tensor 27 infrared spectrophotometer with KBr pellets. ^1^H, ^13^C and 2D NMR spectra were obtained on Bruker AV-600, AVANCE III-500 and 400 MHz spectrometers with SiMe_4_ as an internal standard. Chemical shifts (*δ*) were expressed in ppm with reference to the solvent signals. MS data were recorded on an UPLC-IT-TOF MS. Column chromatography (CC) was performed on either silica gel (200–300 mesh, Qingdao Marine Chemical Co., Ltd., Qingdao, China) or RP-18 silica gel (20–45 *μ*m, Fuji Silysia Chemical Ltd., Japan). Fractions were monitored by TLC on silica gel plates (GF254, Qingdao Marine Chemical Co., Ltd., Qingdao, China), and spots were visualized with Dragendorff’s reagent spray. MPLC was performed using a Buchi pump system coupled with RP-18 silica gel-packed glass columns (15 × 230 and 26 × 460 mm, respectively). HPLC was performed using Waters 1525 pumps coupled with analytical or preparative Sunfire C_18_ columns (4.6 × 150 and 19 × 250 mm, respectively). The HPLC system employed a Waters 2998 photodiode array detector and a Waters fraction collector III.

### Plant Material

Flowers of *E. variegata* Linn and *E. crista*-*galli* Linn were collected in February and April, respectively, 2014 in Simao of Yunnan Province, People’s Republic of China. Leaves and flowers of *Erythrina arborescens* Roxb. Hort. Beng were collected in October 2014 in Jianshui of Yunnan Province. These plant samples were identified by Dr. Chun-Xia Zeng. The voucher specimens (Cai20140207, Cai20140407, Cai20141003 and Cai20141004) have been deposited in the State Key Laboratory of Phytochemistry and Plant Resources in West China, Kunming Institute of Botany, the Chinese Academy of Sciences.

### Extraction and Isolation

The dried and powdered flowers of *E. variegata* (10.0 kg) were extracted with 90% MeOH (25 L) for three times. The extracts were concentrated under reduced pressure, and then dissolved in 2% acetic acid to adjust pH to 2–3 and then partitioned twice with EtOAc. The aqueous layers were basified with NH_3_·H_2_O to adjust pH to 8–9 and then extracted with EtOAc to give a crude alkaloid fraction (110 g). The crude alkaloid was subjected to column chromatography (CC) over silica gel with gradient CHCl_3_-Acetone (1:0 to 1:1) to afford seven fractions (Fr. I–Fr. VII). Fr. I (6.1 g) was divided into 2 subfractions (Fr. I-1–Fr. I-2) by using RP-MPLC eluting with MeOH-H_2_O (50–100%). Fr. I-2 was isolated by preparative C_18_ HPLC column with a gradient of MeOH–H_2_O (60:40–70:30, v/v) to obtain **9** (35 mg) and **11** (41 mg). Fr. II (4.5 g) was separated using C_18_ MPLC column with a gradient of MeOH–H_2_O (40:60–90:40, v/v) to afford **10** (35 mg) and **8** (13 mg). Fr. IV (8.5 g) was fractionated by C_18_ MPLC column with a gradient of MeOH–H_2_O (10:90–90:10, v/v) to give six subfractions (Fr. IV-1–Fr. IV-6). Fr. IV-2 was subjected to a preparative C_18_ HPLC column with a gradient of MeOH–H_2_O (50:50–60:40, v/v) to afford **6** (20 mg) and **5** (32 mg) Fr. IV-4 was subjected to a preparative C_18_ HPLC column with a gradient of MeOH–H_2_O (70:30–80:20, v/v) to give **1** (5 mg). Fr. V (12 g) was chromatographed on a C_18_ MPLC column eluted with a gradient of MeOH–H_2_O (10:80–100:0, v/v) to give six subfractions (Fr. V-1–Fr.V-6). **2** (29 mg) and **3** (21 mg) was obtained from Fr.V-5 using a preparative C_18_ HPLC column with a gradient of MeOH–H_2_O (65:35–25:75, v/v).

Flowers of *E. crista*-*galli* (11 kg) were powdered and extracted with 90% MeOH (25 L) for three times. The extract was concentrated in vacuo to give a brown residue. The crude alkaloid (90 g) were obtained using the same acid–base treatment method described above, and then subjected to column chromatography (CC) over silica gel and eluted with gradient CHCl_3_-Acetone (1:0 to 1:1) to afford four fractions (Fr. I–Fr. IV). Fr. I (12.1 g) was further chromatographed on a C_18_ MPLC column eluted with a gradient of MeOH–H_2_O (50:50–100:0, v/v) to give the two subfractions (Fr. I-1–Fr. I-2). Alkaloid **12** (51 mg) was obtained from Fr. I-1 using a column chromatography (CC) over silica gel and eluted with petroleum ether-acetone (4:1).

Crude alkaloid extract (85.2 g) and (62.5 g) were obtained from the leaves (15.8 kg) and flowers (6.5 kg) of *Erythrina arborescens*, respectively. The crude alkaloid of leaves was divided into nine fractions (Fr. I–Fr. IX). Fr. V (7.1 g) was fractionated by C_18_ MPLC column with a gradient of MeOH–H_2_O (20:80–80:20, v/v) to give three subfractions (Fr. V-1–Fr. V-3). Fr. V-3 was subjected to a preparative C_18_ HPLC column with a gradient of MeOH–H_2_O (60:40–70:30, v/v) to afford **4** (1 mg) and **14** (5 mg). The crude alkaloid of flowers was divided into seven fractions (Fr. I–Fr. VII). **7** was obtained from Fr. II by C_18_ MPLC column with a gradient of MeOH–H_2_O (40:60–100:0, v/v) and then purified by preparative C_18_ HPLC column with a gradient MeOH–H_2_O (50:50–60:40, v/v). Fr. IV (4.5 g) was chromatographed on a C_18_ MPLC column eluted with a gradient of MeOH–H_2_O (20:80–70:30, v/v) to give four subfractions (Fr. IV-1–Fr. IV-4). Fr. IV-3 was further purified by a preparative C_18_ HPLC column with a gradient of MeCN–H_2_O (25:75–35:65, v/v) to afford **13** (20 mg).

**8*α*-(2-oxopropyl)-erythrivarine A** (**1**): white powder; $[\alpha]^{20}_{\text D}$ − 121.2 (*c* 0.2, MeOH); UV (MeOH) *λ*_max_ (log *ε*) 204 (4.26), 239 (3.88) and 290 (3.46) nm; IR (KBr) *ν*_max_ 3441, 2924, 1640, 1503, 1480, 1226, 1100, 1041 cm^−1^; ^1^H (600 MHz) and ^13^C NMR (150 MHz) data (acetone-*d*_6_), see Table 1; positive HRESIMS *m/z* 681.2811 [M+H] ^+^ (calcd. for C_39_H_41_N_2_O_9_, 681.2110).

**8*α*-(2-oxopropyl)-11′-*O*-methyl-erythrivarine A** (**2**): white powder; $[\alpha]^{20}_{\text D}$ −114.1 (*c* 0.1, MeOH); UV (MeOH) *λ*_max_ (log *ε*) 203 (4.12), 238 (3.66) and 289 (3.43) nm; IR (KBr) *ν*_max_ 3441, 1639, 1490, 1234, 1101, 1040 cm^−1^; ^1^H (600 MHz) and ^13^C NMR (150 MHz) data (acetone-*d*_6_), see Table 1; positive HRESIMS *m/z* 695.2968 [M+H]^+^ (calcd. for C_40_H_43_N_2_O_9_, 695.2969).

**8*α*-(2-oxopropyl)-11′-epi-*O*-methyl-erythrivarine A** (**3**): white powder; $[\alpha]^{20}_{\text D}$ −165.2 (*c* 0.1, MeOH); UV (MeOH) *λ*_max_ (log *ε*) 204 (4.22), 238 (3.88) and 289 (3.46) nm; IR (KBr) *ν*_max_ 3441, 2924, 1629, 1503, 1482, 1234, 1100, 1040 cm^−1^; ^1^H (600 MHz) and ^13^C NMR (150 MHz) data (acetone-*d*_6_), see Table 1; positive HRESIMS *m/z* 695.2970 [M+H] ^+^ (calcd. for C_40_H_43_N_2_O_9_, 695.2969).

**8*α*-(2-oxopropyl)-erythrinine** (**4**): white powder; $[\alpha]^{20}_{\text D}$ + 133.0 (*c* 0.2, MeOH); UV (MeOH) *λ*_max_ (log *ε*) 204 (4.71), 239 (3.63) and 289 (3.32) nm; IR (KBr) *ν*_max_ 2930, 1630, 1503, 1489 cm^−1^; ^1^H (600 MHz) and ^13^C NMR (150 MHz) data (acetone-*d*_6_), see Tables 2 and 4; positive HRESIMS *m/z* 370.1651 [M+H]^+^ (calcd. for C_21_H_24_NO_5_, 370.1652).

**Table 2:** ^1^H NMR spectroscopic data for **4**–**8** in acetone-*d*_6_ (*δ* in ppm and *J* in Hz)

Entry	*δ*_H_ (**4**)^a^	*δ*_H_ (**5**)^a^	*δ*_H_ (**6**)^a^	*δ*_H_ (**7**)^b^	*δ*_H_ (**8**)^a^
1	6.55, dd (10.2, 2.4)	6.72, dd (10.2, 2.4)	6.55, dd (10.2, 1.8)	6.60 dd (10.2, 1.9)	6.59, dd (10.2, 2.4)
2	6.01, d (10.2)	6.68, d (10.2)	6.03, d (10.2)	6.10, d (10.2)	6.07, d (10.2)
3	3.79, m	3.85, m	3.92, m	3.97, m	3.85, m
4	2.38, dd (11.4, 5.4)	2.48, dd (11.4,5.6)	2.46, dd (11.4, 5.4)	2.53, dd (11.2, 5.7)	2.45, dd (11.4, 5.4)
	1.65, t (11.4)	1.68, t (11.4)	1.66, t (11.4)	1.78, t (11.2)	1.73, t (11.4)
7	5.67, s	5.63, s	5.64, s	5.67, s	5.71, s
8	4.20, m	3.98, m	4.33, m	4.33, s	4.68, s
10	3.48, dd (13.8, 4.8)	3.38, m	3.39, dd (13.8, 4.2)	3.49, m	3.57, dd (13.2, 4.8)
	2.68, dd (13.8, 6,6)	2.77 (overlap)	3.09, dd (13.8, 4.2)	3.02 dd (12.9, 5.7)	2.92, dd (13.2, 4.8)
11	4.75, m	2.77 (overlap)	4.18, t (4.2)	2.93, m	4.77, m
		2.65, m		264, m	
14	6.70, s	6.72, s	6.74, s	6.84, s	6.73, s
17	7.05, s	6.68, s	6.89, s	6.76, s	7.04, s
3-OCH_3_	3.25, s	3.28, s	3.28, s	3.28, s	3.23, s
11-OCH_3_			3.55, s		
11-OH	4.72, d (4.8)				4.64, br s
15-OCH_3_				3.70, s	
16-OCH_3_				3.97, s	
OCH_2_O	6.01, s	5.92, s	5.96, s		5.95, s
	5.99, s	5.90, s	5.94, s		5.94, s
CH_2_COCH_3_	2.90, dd (15.6, 4.8)	2.85 (overlap)	2.94, overlap		
	2.55, dd (15.6, 4.8)	2.58, dd (16.8, 8.0)	2.55, dd (15.6, 9.0)		
CH_2_COCH_3_	2.13, s	2.13, s	2.12, s		
COOCH_3_				3.78, s	3.72, s

^a1^H NMR recorded in 600 MHz

^b1^H NMR recorded in 400 MH

**8*α*-(2-oxopropyl)-erythraline** (**5**): white powder; $[\alpha]^{23}_{\text D}$ + 80.5 (*c* 0.10, MeOH); UV(MeOH) *λ*_max_ (log *ε*) 201 (4.11), 238 (2.37), 289(1.29) nm; IR (KBr) *ν*_max_ 1712, 1480, 1423 cm^−1^; ^1^H (600 MHz) and ^13^C NMR (150 MHz) data (acetone-*d*_6_), see Tables 2 and 4; positive HRESIMS *m/z* 354.1709 [M+H]^+^ (calcd. for C_21_H_23_NO_4_, 354.1707).

**8*α*-(2-oxopropyl)-11-methoxy-erythraline** (**6**): white powder; $[\alpha]^{20}_{\text D}$ + 95.3 (*c* 0.1, MeOH); UV (MeOH) *λ*_max_ (log *ε*) 204 (4.76), 238 (3.74) and 288 (3.48) nm; IR (KBr) *ν*_max_ 1631, 1504, 1483 cm^−1^; ^1^H (600 MHz) and ^13^C NMR (150 MHz) data (acetone-*d*_6_), see Tables 2 and 4; positive HRESIMS *m/z* 384.1806 [M+H]^+^ (calcd. for C_22_H_26_NO_5_, 384.1805).

**8*α*-carbomethoxyerysotrine** (**7**): white powder; $[\alpha]^{22}_{\text D}$ + 74.0 (*c* 0.21, CH_3_OH); UV (CH_3_OH) *λ*_max_ (log *ε*) 203 (3.79), 225 (3.45) and 277 (3.05) nm; ^1^H (400 Hz) and ^13^C (125 Hz) NMR data (acetone-*d*_6_), Tables 2 and 4; Positive ESIMS *m/z* 394 [M+Na]^+.^, HRESIMS *m/z*. 394.1626 [M+Na]^+^; (calcd. for C_21_H_25_NO_5_Na, 394.1625).

**8*α***-**carbomethoxyerythrinine** (**8**): white powder; $[\alpha]^{20}_{\text D}$ + 118.3 (*c* 0.1, MeOH); UV (MeOH) *λ*_max_ (log *ε*) 205 (4.69), 239 (3.79) and 289 (3.51) nm; IR (KBr) *ν*_max_ 2923, 1630, 1503, 1488 cm^−1^; ^1^H (600 MHz) and ^13^C NMR (150 MHz) data (acetone-*d*_6_), see Tables 2 and 4; positive HRESIMS *m/z* 372.1444 [M+H]^+^ (calcd. for C_20_H_22_NO_6_, 372.1443).

**8*α*-acetatemethoxyerythraline** (**9**): white powder; $[\alpha]^{20}_{\text D}$ + 171 (*c* 0.1, MeOH); UV (MeOH) *λ*_max_ (log *ε*) 204 (4.78), 239 (3.88) and 290 (3.52) nm; IR (KBr) *ν*_max_ 1628, 1501, 1489 cm^−1^; ^1^H (600 MHz) and ^13^C NMR (150 MHz) data (acetone-*d*_6_), see Tables 3 and 4; positive HRESIMS *m/z* 370.1652 [M+H]^+^ (calcd. for C_21_H_24_NO_5_, 370.1653).

**Table 3:** ^1^H NMR spectroscopic data for **9–14** in acetone-*d*_6_ (*δ* in ppm, *J* in Hz)

Entry	*δ*_H_(**9**)^a^	*δ*_H_(**10**)^a^	*δ*_H_(**11**)^a^	*δ*_H_(**12**)^a^	*δ*_H_ (**13**)^b^	*δ*_H_ (**14**)^a^
1	6.54, dd (10.2, 2.4)	6.55, dd (10.2, 2.4)	6.56, dd (10.2, 1.8)	6.57, dd (10.2, 2.4)	6.60, d (10.2)	6.58, dd (10.2, 2.2)
2	6.00, d (10.2)	5.99, d (10.2)	6.04, d (10.2)	6.05, d (10.2)	6.14, d (10.20	6.11, d (10.2)
3	3.8, m	3.77, m	3.91, m	4.01, m	4.09, m	3.92, m
4	2.48, dd (11.4, 5.4)	2.38, overlap	2.46, dd (11.4, 5.4)	2.50, m	2.63, dd (10.9, 5.7)	2.66, dd (11.4, 5.5)
	1.69, t (11.4)	1.64, t (11.4)	1.66, t (11.4)	1.69, t (10.8)	1.78, t (10.9)	1.77, t (11.4)
7	5.66, s	5.70, s	5.69, s	5.64, s	5.68, s	5.72, s
8	3.94, m	4.16, m	4.31, m	4.05, m	4.14, m	4.07, m
10	3.40, m	3.50, dd (12.0, 5.4)	3.39, dd (13.8, 4.2)	3.37, m	3.49, m	3.53, m
	2.75, m	2.65, dd (12.0, 6.6)	3.06, dd (13.8, 4.2)	2.95, m	3.16, m	3.03, m
11	2.84, overlap	4.77, m	4.19, t (4.2)	2.96, m	3.03, m	2.98, m
	2.65, overlap			2.57, m	2.72, overlap	2.84, m
14	6.70, s	6.69, s	6.90, s	6.83, s	6.84, s	6.73, s
17	6.68, s	7.05, s	6.74, s	6.81, s	6.78, s	6.74, s
3-OCH_3_	3.25, s	3.23, s	3.29, s	3.28, s	3.31, s	3.30, s
11-OCH_3_			3.55, s			
11-OH		4.60, d (5.4)				
15-OCH_3_				3.78, s	3.68, s	
16-OCH_3_				3.67, s	3.79, s	
OCH_2_O	5.91, s	5.95, s	5.96, s			5.96, s
	5.89, s	5.94, s	5.94, s			5.95, s
CH_2_COO–	2.68, dd (15.0, 4.8)	2.73, dd (15.0, 4.8)	2.81, dd (15, 4.2)	2.72, dd (15.0, 4.8)	2.76, overlap	2.74, dd (16.8, 5.8)
	2.39, dd (15.0, 7.8)	2.36, overlap	2.36, dd (15, 6.0)	2.41, dd (15.0, 7.8)	2.51, dd (16.5,2.8)	2.51, dd (16.8, 1.8)
CH_2_COOCH_3_	3.61, s	3.60, s	3.62, s	3.63, s		

^a1^H NMR recorded in 600 MHz

^b1^H NMR recorded in 400 MH

**Table 4:** ^13^C NMR spectroscopic data from **4** to **14** in acetone-*d*_6_ (*δ* in ppm)

Entry	*δ*_C_ (**4**)^a^	*δ*_C_ (**5**)^a^	*δ*_C_ (**6**)^a^	*δ*_C_(**7**)^b^	*δ*_C_ (**8**)^a^	*δ*_C_ (**9**)^a^	*δ*_C_ (**10**)^a^	*δ*_C_ (**11**)^a^	*δ*_C_ (**12**)^a^	*δ*_C_(**13**)^b^	*δ*_C_(**14**)^a^
1	125.4 d	125.6 d	125.7 d	125.3 d	125.2 d	125.4 d	125.3 d	125.5 d	125.6 d	125.1 d	125.0 d
2	133.1 d	133.1 d	133.1 d	134.0 d	134.2 d	133.1 d	133.2 d	133.1 d	133.2 d	134.1 d	134.1 d
3	76.9 d	77.0 d	76.8 d	76.9 d	76.8 d	76.8 d	76.9 d	76.7 d	77.0 d	76.6 d	76.5 d
4	43.3 t	43.8 t	42.7 t	43.3 t	43.1 t	43.8 t	43.6 t	42.7 t	43.4 t	42.3 t	42.7 t
5	69.3 s	69.3 s	68.6 s	69.4 s	69.7 s	69.6 s	69.7 s	68.9 s	68.9 s	69.1 s	69.7 s
6	142.3 s	142.6 s	142.4 s	144.4 s	144.2 s	142.7 s	142.4 s	142.4 s	142.9 s	143.1 s	143.0 s
7	127.6 d	127.6 d	127.9 d	121.7 d	122.4 d	126.9 d	127.1 d	127.3 d	126.7 d	125.2 d	125.6 d
8	67.7 d	65.1 d	65.8 d	70.5 d	73.2 d	66.1 d	68.5 d	66.4 d	64.4 d	63.9 d	65.4 d
10	52.6 t	43.9 t	45.2 t	44.4 t	53.1 t	44.2 t	53.0 t	45.4 t	42.6 t	40.8 t	42.3 t
11	64.7 d	26.0 t	74.8 d	24.6 t	65.1 d	26.0 t	64.8 t	74.7 d	24.6 t	24.1 t	25.5 t
12	132.2 s	129.4 s	130.1 s	128.0 s	133.3 s	129.4 s	133.5 s	130.0 s	127.7 s	126.8 s	128.7 s
13	133.4 s	134.0 s	133.0 s	132.2 s	131.9 s	133.9 s	132.2 s	132.9 s	132.7 s	130.9 s	132.2 s
14	105.9 d	106.5 d	105.9 d	110.6 d	105.8 d	106.4 d	105.8 d	105.7 d	110.6 d	110.4 d	106.4 d
15	147.3 s	146.7 s	148.0 s	148.2 s	147.6 s	146.6 s	147.3 s	147.8 s	148.9 s	148.5 s	147.0 s
16	147.2 s	147.0 s	147.2 s	149.1 s	147.5 s	146.9 s	147.3 s	147.1 s	148.1 s	149.4 s	147.5 s
17	107.4 d	109.4 d	109.0 d	113.1 d	108.1 d	109.3 d	107.2 d	108.8 d	113.0 d	113.1 d	109.5 d
3-OCH_3_	56.2 q	56.3 q	56.3 q	56.0 q	56.3 q	56.1 q	56.1 q	56.2 q	56.0 q	55.9 q	56.4 q
11-OCH_3_			57.9 q					57.7 q		56.0 q	
15-OCH_3_				56.1 q					56.1 q	56.3 q	
16-OCH_3_				56.1 q					55.9 q		
OCH_2_O	101.7 t	101.7 t	101.9 t		101.9 t	101.5 t	101.7 t	101.7 t			101.9 t
CH_2_COCH_3_	47.8 t	48.1 t	47.4 t							34.9 t	35.4 t
CH_2_COCH_3_	207.9 s	207.7 s	207.7 s							172.3 s	172.3 s
CH_3_COCH_3_	30.8 q	30.9 q	30.8 q								
(CH_2_)COOCH_3_						39.5 t	39.3 t	38.8 t	39.3 t		
(CH_2_)COOCH_3_				172.1 s	172.2 s	172.5 s	172.4 s	172.3 s	172.9 s		
(CH_2_)COOCH_3_				52.0 q	52.1 q	51.5 q	51.5 q	51.5 q	51.6 q		

^a13^C NMR recorded in 150 MHz

^b13^C NMR recorded in 125 MHz

**8*α*-acetatemethoxyerythrinine** (**10**): white powder; $[\alpha]^{20}_{\text D}$ + 89.4 (*c* 0.1, MeOH); UV (MeOH) *λ*_max_ (log *ε*) 204 (4.65), 238 (3.68) and 290 (3.33) nm; IR (KBr) *ν*_max_ 2924, 1628, 1488 cm^−1^; ^1^H (600 MHz) and ^13^C NMR (150 MHz) data (acetone-*d*_6_), see Tables 3 and 4; positive HRESIMS *m/z* 386.1599 [M+H]^+^ (calcd. for C_21_H_24_NO_6_, 386.1598).

**8*α*-acetatemethoxy-11*β*-methoxyerythraline** (**11**): white powder; $[\alpha]^{20}_{\text D}$ + 154.2 (*c* 0.2, MeOH); UV (MeOH) *λ*_max_ (log *ε*) 204 (4.62), 238 (3.74) and 289 (3.41) nm; IR (KBr) *ν*_max_ 1630, 1503, 1484 cm^−1^; ^1^H (600 MHz) and ^13^C NMR (150 MHz) data (acetone-*d*_6_), see Tables 3 and 4; positive HRESIMS *m/z* 400.1758 [M+H]^+^ (calcd. for C_22_H_26_NO_6_, 400.1757).

**8*α*-acetatemethoxyerysotrine** (**12**): white powder; $[\alpha]^{20}_{\text D}$ + 113.3 (*c* 0.1, MeOH); UV (MeOH) *λ*_max_ (log *ε*) 204 (4.72), 239 (3.66) and 288 (3.51) nm; IR (KBr) *ν*_max_ 1628, 1503, 1488 cm^−1^; ^1^H (600 MHz) and ^13^C NMR (150 MHz) data (acetone-*d*_6_), see Tables 3 and 4; positive HRESIMS *m/z* 386.1964 [M+H]^+^ (calcd. for C_22_H_28_NO_5_, 386.1965).

**8*α*-acetylerythsotrine** (**13**): white powder; C_21_H_25_NO_5_; $[\alpha]^{22}_{\text D}$ + 5.2 (c 0.18, CH_3_OH); UV (CH_3_OH) *λ*_max_ (log *ε*) 203 (3.90), 229 (3.56) and 282 (2.93) nm; ^1^H (400 Hz) and ^13^C (125 Hz) NMR data (acetone-*d*_6_), Tables 3 and 4; Positive ESIMS *m/z* 394 [M+Na]^+.^, HRESIMS *m/z*. 394.1628 [M+Na]^+^; (calcd. for C_21_H_25_NO_5_Na, 394.1625).

**8*α*-acetylerythraline** (**14**): white powder; $[\alpha]^{20}_{\text D}$ + 105 (*c* 0.1, MeOH); UV (MeOH) *λ*_max_ (log *ε*) 204 (4.71), 249 (3.72) and 289 (3.62) nm; IR (KBr) *ν*_max_ 3341, 1639, 1503, 1442 cm^−1^; ^1^H (600 MHz) and ^13^C NMR (150 MHz) data (acetone-*d*_6_), see Tables 3 and 4; positive HRESIMS *m/z* 378.1313 [M+H]^+^ (calcd. for C_20_H_22_NO_5_, 378.1314).

### Cytotoxicity

The human A-549 lung cancer, SGC-7901 gastric cancer, and HeLa cell lines were used in the cytotoxic assay. These cells were grown in DMEM media (HyClone, USA) supplemented with 10% fetal bovine serum (HyClone, USA) at 37 °C in 5% CO_2_. The cytotoxicity of all alkaloids were determined based on the MTT method in 96-well microplates. In short, 100 *µ*L adherent cells were seeded into each well and incubated for 12 h before the addition of the test alkaloids/drug. At the same time, the suspended cells were seeded at an initial density of 1 × 10^5^ cells/mL just before the addition of the alkaloids/drug. Each tumor cell line was exposed to a test compound at concentration 20 μM in DMSO in triplicate for 48 h, with camptothecin as the positive control. After treatment, cell viability was assessed.

### Hypoglycemic Activity

3T3-L1 myoblasts cells were purchased from American Type Culture Collection (Manassas, VA). Cells were maintained in DMEM supplemented with 10% FBS or CS (for 3T3-L1 cells), 100 units/ml penicillin and 100 mg/ml streptomycin in 10 cm diameter dishes in a humidified atmosphere of 95% air and 5% CO_2_ at 37 °C. Cells were maintained in continuous passages by trypsinization of subconfluent cultures and fed fresh medium every 48 h. For differentiation, L6 myoblasts were transferred to DMEM with 2% FCS in tissue culture plates for 5-6 days, 3T3-L1 cells were exposed to 0.5 mM IBMX, 1 mM dexamethasone, 1 mM rosiglitazone and 1 mg/mL insulin for 3 days, and 1 mg/ml insulin for the other day. For glucose uptake assay, cells were serum starved for 4 h in 96-well plates, followed by incubated with insulin and alkaloids **1**–**14** for 24 h. Finally, the supernatants of cultured cells were collected and subjected to glucose assay using a commercially kit. The quantified values were normalized based on the results of the MTS assay.
